# Early rehabilitation in sepsis: a prospective randomised controlled trial investigating functional and physiological outcomes *The i-PERFORM Trial *(Protocol Article)

**DOI:** 10.1186/1471-2253-11-21

**Published:** 2011-10-31

**Authors:** Geetha Kayambu, Robert J Boots, Jennifer D Paratz

**Affiliations:** 1 Burns, Trauma & Critical Care Research Centre, School of Medicine, The University of Queensland, Brisbane QLD 4029, Australia; 2 Department of Intensive Care Medicine, The Royal Brisbane and Women's Hospital, Brisbane QLD 4029, Australia

## Abstract

**Background:**

Patients with sepsis syndromes in comparison to general intensive care patients can have worse outcomes for physical function, quality of life and survival. Early intensive care rehabilitation can improve the outcome in general Intensive Care Unit (ICU) patients, however no investigations have specifically looked at patients with sepsis syndromes. The 'i-PERFORM Trial' will investigate if early targeted rehabilitation is both safe and effective in patients with sepsis syndromes admitted to ICU.

**Methods/Design:**

A single-centred blinded randomized controlled trial will be conducted in Brisbane, Australia. Participants (n = 252) will include those ≥ 18 years, mechanically ventilated for ≥ 48 hours and diagnosed with a sepsis syndrome. Participants will be randomised to an intervention arm which will undergo an early targeted rehabilitation program according to the level of arousal, strength and cardiovascular stability and a control group which will receive normal care.

The primary outcome measures will be physical function tests on discharge from ICU (The Acute Care Index of Function and The Physical Function ICU Test). Health-related quality of life will be measured using the Short Form-36 and the psychological component will be tested using The Hospital Anxiety and Depression Scale. Secondary measures will include inflammatory biomarkers; Interleukin-6, Interleukin-10 and Tumour Necrosis Factor-α, peripheral blood mitochondrial DNA content and lactate, fat free muscle mass, tissue oxygenation and microcirculatory flow.

**Discussion:**

The 'i-PERFORM Trial' will determine whether early rehabilitation for patients with sepsis is effective at improving patient outcomes with functional and physiological parameters reflecting long and short-term effects of early exercise and the safety in its application in critical illness.

**Trial Registration:**

Australia and New Zealand Clinical Trials Register (ANZCTR): ACTRN12610000808044

## Background

Critical Illness and trauma are the primary sources of intensive care unit admissions. An estimated 2-11% of these patients require prolonged stays in the ICU which accounts for 25-45% of total ICU days [1]. Survivors of intensive care, especially those with prolonged admissions, may exhibit severe psychological and physical problems [2] and have a lower health-related quality of life up to one year following discharge from the hospital [3]. Almost a quarter of these patients are either admitted with or develop sepsis i.e. a severe, specific inflammatory response to infection, during the course of their stay in the ICU with major immediate and long-term effects on morbidity and mortality [4]. Patients with sepsis can have a worse outcome both in terms of overall functioning and mortality rates [5]. There is evidence that specific decreases in muscle mass and muscle force occur in sepsis syndromes caused by a variety of mechanisms [6]. This has major effects on the health and productivity of survivors as well as ultimately impact on the availability of ICU and hospital beds, surgical waiting lists, health costs and society.

**Figure 1:**
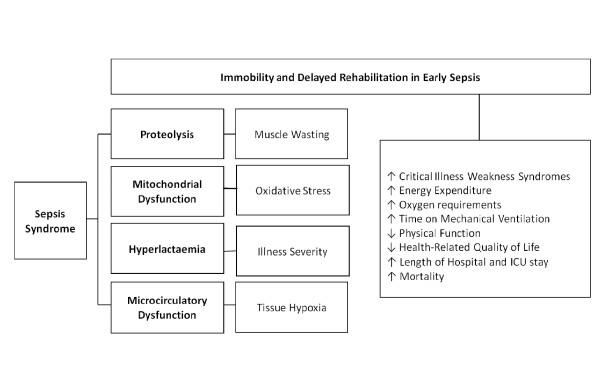
**Impact of delayed ICU rehabilitation in early sepsis**. This diagram illustrates the sequential impact of delayed rehabilitation for patients with sepsis as a result of the inflammatory processes and the detrimental short and long-term outcomes.

Hypothesising that light to moderate exercise can partially reverse this condition without causing harm to critically ill patients, several exercise trials conducted in general intensive care patients have indicated positive findings that early intervention with exercise is able to prevent critical illness weakness syndromes, loss of muscle mass, decrease duration of mechanical ventilation, length of hospital and ICU stay and improve overall quality of life [7]. Specifically however, it is important to investigate early rehabilitation in patients with sepsis syndromes as the inflammatory process in sepsis can aggravate and accelerate the rate of muscle wasting in addition to the immobility associated with the disease in its early stages. It is equally important to ensure that early exercise in sepsis does not result in oxidative stress or cause substantial tissue injury potentially worsening the inflammatory reaction. The physiological reasons for improvement or adverse effects require investigation (Figure 1).

### Sepsis Syndrome

Sepsis is a systemic inflammatory response associated with an infectious insult. It is the leading cause of death in critically ill patients and is often associated multi organ failure [8]. The inflammatory cytokines associated with sepsis such as Interleukin-6 (IL-6), Interleukin-10 (IL-10) and Interleukin-1β (IL-1β) are found to be correlated with the severity of the disease, the evolution of organ failure as measured by the SOFA score and mortality [9]. Physiological responses to inflammation as described in Table 1 in addition to the presence of a suspected or proven infection has been an internationally accepted definition for "Sepsis" [8,10]. Increasing sepsis severity correlates with increasing mortality, rising from 25-30% for severe sepsis to 40-70% for septic shock [11].

**Table 1:** Criteria for SIRS and Sepsis syndromes (modified from Dellinger et al [57])

Syndrome	Criteria	Mortality
Systemic inflammatory response syndrome (SIRS) any two or more of the following criteria	▪ HR>90 bpm ▪ RR>20/min or PaCO_2_< 32 mmHg ▪ WCC< 4 × 10^9 ^cells/L or > 12 × 10^9^ cells/L or > 10% immature bands 12,000 ▪ T>38°C or <36°C	

Sepsis	▪ SIRS + proven infection	15%

Severe sepsis	▪ Sepsis + failure of one or more organs	20%

Septic shock	▪ Severe sepsis + cardiovascular failure despite adequate fluid resuscitation (usually SABP <90 mmHg or >40 mmHg from baseline)	45%

HR; Heart rate, RR; Respiratory Rate; WCC; White Cell Count; T; Temperature, SIRS; Systemic Inflammatory Response Syndrome, SABP; Systolic Arterial Blood Pressure.

### Systemic Inflammation and Proteolysis

The aggressive inflammatory process that occurs during sepsis also affects muscle force, muscle mass and ultimately physical function [12,13]. Persistent elevation of circulating levels of Interleukin-6 (IL-6) have been known to infiltrate myocytes with inflammatory factors such as prostaglandins leading to proteolysis, myocyte degeneration, and muscle atrophy [14]. Tumour Necrosis factor-α (TNF-α) overexpression in sepsis is linked to the development of cachexia [15] through endothelial dysfunction, leading to myocyte apoptosis, reduction in skeletal muscle mass, weakness and myopathy. It has been hypothesised that the prevention of excessive release of pro-inflammatory cytokines [15], and activation of proteolytic pathways leading to limitation of free-radical generation [16] may inhibit the catabolic skeletal muscle changes in sepsis [17] and thus critical illness weakness syndromes.

### Inflammatory Biomarkers and Exercise

Interleukin-10 (IL-10); an anti-inflammatory cytokine is thought to inhibit proteolysis [18] while low levels have been postulated to lead to excessive inflammation and muscle damage [19]. IL-10 is known to attenuate the synthesis of TNF-α surface receptor and its suppressive effects may be beneficial in pathology that results from inflammatory dysregulation such as in sepsis.

A number of studies [20-22] in healthy and other diseased populations; such as in chronic heart failure, have shown that aerobic and resisted exercise alters pro-inflammatory cytokines specifically decreasing IL-6 and TNF-α and increasing IL-10. Simple physical exercises such as repetitive passive muscle stretches have been shown to decrease pro-inflammatory cytokine (IL-6), increase anti-inflammatory cytokine (IL-10) and improve the IL-10/TNF-α ratio in chronic critically ill patients [23]. The use of electrical muscle stimulation on major muscle groups has been shown to attenuate the production of TNF-α [24]. It is therefore important to investigate the effect of exercise on IL-10 in sepsis.

Our pilot study tested 20 patients with sepsis syndromes randomised into a treatment (proactive rehabilitation) and a control group. The trial demonstrated significant decreases (-7.2%) in the percentage fat free mass in the control group with no decreases in the intervention group. Significant increases in IL-10 (mean difference 12.1 [SE +/- 2.1], p < 0.01) were found in the treatment group. Clinically relevant findings from this study indicate early exercise reduces loss of muscle mass in sepsis [25].

### Oxidative stress

Oxygen-derived free radicals play an important role in the development and progression of disease in critically ill patients resulting in increases in the level of Reactive Oxygen Species (ROS) [26] or decreases in antioxidant defences [27] causing oxidative stress [28]. ROS can play a pivotal role in stimulating the inflammatory system by causing an increase in cytokines (e.g. Interleukins and TNF-α). Both cytokines and ROS can enter the circulation and mediate systemic inflammatory responses linked with clinical conditions [29] which are inter-related in causing muscle proteolysis resulting in the induction of sepsis-induced myopathy [6].

In patients with sepsis, antioxidant depletion has been found to be associated with mitochondrial dysfunction where oxidative stress generates bioenergetic failure [30] which may affect changes in mitochondrial DNA (mtDNA) quantity [31], as well as increase mutations or deletions. This has been hypothesised to be part of the mechanism underlying multiple organ failure and death [32,33].

Oxidative stress also acts as an atrophic stimulus in an unloaded muscle [34] promoting wasting by modifying redox-sensitive processes in its muscle fibres such as during periods of disuse in locomotor skeletal muscles [35] and the unloaded diaphragm during prolonged mechanical ventilation [36,37]. Currently, however, there is no published data on the effects of exercise on oxidative stress on the critically ill population particularly in sepsis.

### Illness Severity

Hyperlactaemia is a marker of illness severity in sepsis [38,39]. During early sepsis, perturbation of glycolytic mechanisms can occur. In septic shock, high concentrations of circulating adrenaline can derive large quantities of lactate out of skeletal muscles, overwhelming lactate clearance mechanisms with resultant, hyperlactaemia [40,41]. The effect of light exercise on lactate clearance mechanisms in early sepsis remains to be investigated.

### Microcirculation and Muscle Oxygenation

Sepsis impairs microcirculatory function causing tissue hypoxia [42]. This, combined with blood flow diversion from less important tissues to vital organs [43] promotes decreased muscular oxygen extraction [44]. However, the use of electrical stimulation [45] on major muscle groups and a physical exercise rehabilitation program in chronic heart failure patients [46] have demonstrated short-term beneficial systemic effects on microcirculation. The effect of early activity on microcirculation in patients with sepsis should be further investigated.

### Early Rehabilitation may Modulate Detrimental Effects of Sepsis

The roles of pro-inflammatory cytokines, mitochondrial changes and muscle proteolysis in causing sepsis-induced myopathy in critically ill patients are hypothesised from animal models [6,14]. It is postulated that exercise can modulate cytokine levels [47], ROS production [50] and ATP levels [49] in patients with sepsis. In conjunction with an early mobility protocol, ambulation during mechanical ventilation in the early stages of critical illness is becoming widely practiced [50-52], especially with interruption of sedation [53-55]. Conservatively, simple passive movements [56] and neuromuscular electrical stimulation [45] as a precursor to active mobilisation can induce microcirculatory changes that may attenuate the anti-inflammatory effects in early sepsis.

Overall, early rehabilitation in critical illness seems to show reduced time on the ventilator, improved quality of life and reduced hospital and ICU stay [7] but this is not specific to sepsis. Furthermore, the short-term systemic effects of exercise for ICU patients remain highly speculative and debatable. The impact of exercise in the early stages of critical illness on effects such as oxidative stress and microcirculatory alterations have been unexplored and speculated from other populations. Exploring these physiological factors in response to exercise can contribute to determining the safety of early rehabilitation in sepsis.

### Hypotheses and Aims

The primary research hypothesis of the "i-PERFORM Trial" is that patients with defined sepsis syndromes [57] in the ICU who participate in an early targeted rehabilitation program will have improved physical function and an improved quality of life. The secondary hypotheses is that there will be systemic effects underlying primary improvements which will include; increased fat free mass; decreased pro-inflammatory and increased anti-inflammatory cytokines; increased peripheral blood mitochondrial DNA content and reduced blood lactate levels; and improved tissue oxygenation and microcirculation flow.

## Methods/Design

### Methods and Design

**Figure 2:**
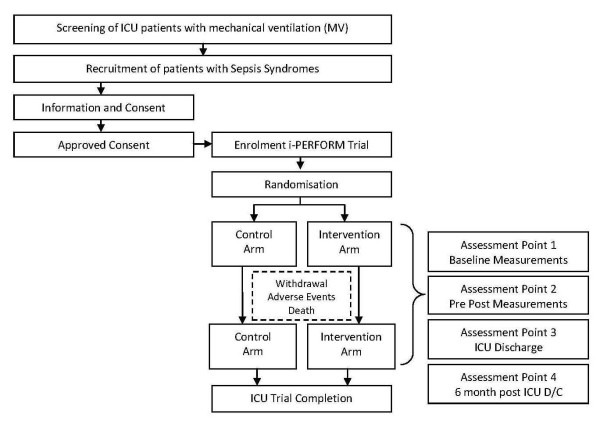
**Research methodology sequence diagram**. The inclusion criteria are as follows: Age ≥ 18 yrs and remain ventilated for ≥ 48 hours; present with documented sepsis or high clinical suspicion of sepsis. Patients with head injuries, burns, spinal injuries, and multiple fractured lower limbs, those with septic shock unresponsive to treatment, moribund or with expected mortality within 48 hours will be excluded.

The i-PERFORM Trial is a prospective double blinded randomised controlled trial (RCT) in patients with sepsis syndromes randomised into an early rehabilitation intervention arm or a control arm during the course of ICU stay. All outcomes will be measured by a blinded assessor from the research team. This study is being conducted in a quaternary level general Intensive Care Unit at the Royal Brisbane and Women's Hospital (RBWH), Brisbane, Queensland, Australia. The Human Research Ethics Committee at RBWH and the Medical Research Ethics Committee at The University of Queensland have approved this study. The study protocol is registered with the Australian New Zealand Clinical Trials Registry (ANZCTR). Figure 2 illustrates the methodology design for this RCT through recommendations from CONSORT (Consolidated Standards of Reporting Trials) Statement.

### Inclusion Criteria

To be eligible for recruitment in the study, participants must be aged 18 years and above; ventilated for ≥ 48 hours; diagnosed with a sepsis syndrome or have a high clinical suspicion of sepsis [57]. Patients with head injuries, burns, spinal injuries, and multiple fractured lower limbs requiring specific rehabilitation regimes and patients with septic shock who are unresponsive to maximal treatment and those who are moribund or have an expected mortality within 48 hours will be excluded.

### Recruitment, Randomisation and Blinding

Eligible patients will be identified and consent will be sought by the chief and principal investigators, with next of kin or substitute decision maker. Participants recruited will be randomized into control and intervention arms using computer generated randomization; http://www.randomization.com/. The randomization sequence will be generated and re-identifiable serial numbers will be assigned by a research personnel not involved in the study. They will be concealed from consent designee research staff for group allocation to prevent selection bias and protected by an electronic password. Participants, substitute decision makers and outcome assessors will be blinded to group allocation to avoid influence over treatment effect. Blinding of the treating physiotherapist and health care providers cannot be ensured due to the nature of the research intervention.

### Demographics

Patient demographic information will be collected at baseline (recruitment) and ICU and hospital discharge and will include; age, gender, primary reason for readmission to ICU, Acute Physiological and Chronic Health Evaluation II (APACHE II)[58], Sequential Organ Failure Assessment (SOFA) score [59], Charlson Comorbidity Index [60], time on mechanical ventilation, length of ICU and hospital stay, readmissions to ICU and 90-day mortality.

### Intervention Arm

Participants randomised to the intervention arm of the trial will undergo a specific targeted rehabilitation program prescribed by the treating ICU physiotherapist for 30 minutes, one to two times daily until discharge from the ICU within 48 hours of the diagnosis of sepsis (Early Intervention Arm). The rehabilitation program will comprise of passive or active range of motion exercises, resistive exercises, electrical muscle stimulation to major muscle groups, leg or arm ergometry, sitting out of bed, tilt table therapy and ambulation with assistance. The rehabilitation strategy for the participants in the intervention arm has been carefully planned with consideration for different levels of cardiovascular stability, ability of the patient to co-operate, and existing evidence regarding rehabilitation techniques and mobilisation strategies in intensive care. It will be administered and progressed by the discretion of the physiotherapist, according to individual acuity of illness and level of co-operation as based on the Ramsay sedation score which will account for the intervention progression stages. If the patient deteriorates, a lower level of activity will be given. Table 2 summarises the intervention strategies.

**Table 2:** Intervention Strategies for early targeted rehabilitation for the critically ill.

Ramsay Sedation Scale	Stages of exercise progression	Conditions	Type of Intervention	Recommended Exercise Prescription
6-4	Stage 1	Sedated	Passive Range of Motion with stretch reflex to upper and lower limbs	30 Mins/day
			Electrical Muscle Stimulation to major muscle groups	30 Mins/day
3-2	Stage 2	Inotropic Dependence	Active Range of Motion/Lightly resisted with upper and lower limb	10 Mins × 2/day
			Electrical Muscle Stimulation to major muscle groups	30 Mins/day
			Sitting up in Bed with assistance as tolerated	30 Mins × 2/day
			Sitting on Edge of Bed	10 Mins × 2/day
2	Stage 3	Weight	Active Range of Motion/Lightly resisted with upper and lower limb	10 Mins × 2/day
		Bearing	Electrical Muscle Stimulation to major muscle groups	30 Mins/day
		Muscle Strength	Sitting On Edge of Bed	10 Mins × 2/day
		Grade < 3	Sitting Out of Bed with assistance	60 Mins × 2/day
			Lower Limb Ergometry	15 Mins × 2/day
			Tilt Table Therapy	30 Mins/day
2	Stage 4	Weight	Active Range of Motion/Lightly resisted with upper and lower limb	10 Mins × 2/day
		Bearing	Electrical muscle stimulation to major muscle groups	30 Mins/day
		Muscle Strength	Sitting On Edge of Bed	10 Mins/day
		Grade > 3	Sitting Out of Bed	30 Mins/day
			Upper Limb Ergometry (low level resistance)	15 Mins/day
			Lower Limb Ergometry	15 Mins × 2/day
			Ambulation with assistive device and therapist	30 Mins × 2/day

The Intervention strategies act as a guide for the execution of early exercises for the patients with sepsis syndromes based on the level of alertness according to the Ramsay Sedation Scale

### Control Arm

Participants allocated to the control arm will receive standard ICU care. The participants randomised to this group will not receive active rehabilitation from the research team, i.e. will not be given targeted exercises early in their disease process upon recruitment. However, they will continue to receive simple and less regular mobilisation activities from other sources, as part of the usual ICU care such as sitting out of bed or ambulation immediately prior to discharge from the unit. The time involved in these activities and the type of activities performed will be recorded and compared between groups.

### Safety and Withdrawal criteria

A safety audit will be completed on patients in the rehabilitation group to monitor any adverse events [61] during rehabilitation. Data will be gathered from the patient's IntelliVue bedside monitor MP70 (Phillips) every 10 seconds and printed out for 10 minutes prior, during and post rehabilitation. The intra-arterial line will be zeroed 10 minutes prior to exercise. A withdrawal criterion with a checklist of adverse events will be used for the clinical decision of withdrawal or modification of exercise intervention.

### Primary Outcome Measures

All primary outcomes will be measured at baseline (recruitment) and at ICU discharge and quality of life at 6 months post hospital discharge. The primary outcome measures will report the level of physical functioning and quality of life (long-term effects) of the participants. Physical function will be measured using the Acute Care Index of Function (ACIF) and the Physical Function ICU Test (PFIT).

The ACIF will essentially rate simple physical function (transfer bed to chair, sit to stand independently) of the patient on leaving the ICU [62]. The PFIT is a newly developed clinical tool for testing functional strength and endurance capacity in ICU patients. It consists of a battery of tests assessing endurance, muscle strength, cardiovascular capacity and functional ability which are domain representative of physical function and specific for the use of higher functional assessment in the critically ill [63]. These have been chosen as corresponding measuring instruments as they have demonstrated reliability, validity and responsiveness in the ICU population [63,64].

Quality of life will be measured using the Short Form-36 (SF-36) Health Survey Questionnaire. Information from the next of kin will be used to provide proxy scores which have been demonstrated to have good reliability and validity [65].

The constructs of psychological well being will be measured using the anxiety subscale of the Hospital Anxiety and Depression Scale (HADS) as psychological wellbeing is known to be highly correlated to functional physical outcomes [66].

### Secondary Outcome Measures

The secondary outcome measures will report on the physiological factors (short-term effects) illustrating underlying mechanisms of early intervention undertaken in the study. Blood samples (8 ml) will be obtained from the patients' arterial line, for the analysis of cytokines, blood lactate and mtDNA levels pre and post intervention during the trial. All blood samples taken will be centrifuged (Spintron GT-25E/LL, Australia) for 15 minutes at 3000 rpm, within 20 minutes of collection. Plasma from the whole blood for the cytokine analysis and peripheral blood mononuclear cells (PBMC) forming the buffy coat pellet for DNA isolation will be stored at -80°C.

#### Inflammatory Biomarkers

Biomarkers will be measured before intervention and 30 minutes post intervention daily (week 1) and thereafter twice weekly (till ICU discharge). The acute inflammatory response of early exercise in sepsis will be measured by changes in levels of pro-inflammatory cytokines (IL-6 and TNF-α) and anti-inflammatory cytokine (IL-10). IL-6, IL-10 and TNF-α will be measured from plasma samples with the Milliplex cytokine panels from Millipore (Billerica, MA, USA) using a Luminex 100 assay, with inter and intra-assay CV < 7%.

#### Muscle Mass

Fat free mass will account for muscle mass (lean tissue) measured at baseline (week 0) and weekly thereafter (till ICU discharge) using the Multi-Frequency Bioelectrical Impedance Spectroscopy (BIS) Machine (ImpediMed SFB7, ImpediMed Ltd, Brisbane, Australia). Measurements will be taken at a standardised time of the day as practicable for validity [67] and at standardised anatomical landmarks for reproducibility of results [68]. Pairs of gel electrodes will be placed on the hand and foot on the right side of the body with the participant positioned in supine. ICU monitoring will continue during measurement without interference [69]. An estimate will be made of the critically ill patients' fluid balance. Urinary catheters will be emptied, prior to measurement. If subjects are known to have ascites, a pleural effusion or a renal replacement therapy, measurements will not be done. Percentage fat free mass will be measured in triplicate and analysed to reflect if proteolysis had occurred and resulted in the loss of muscle mass [70].

#### Oxidative Stress Markers

Oxidative stress will be determined through changes in mitochondrial DNA levels. Mitochondrial DNA copy number in the PBMC will be measured by determining relative amounts of mitochondrial to nuclear DNA using quantitative real-time PCR. Mitochondrial DNA (mtDNA) levels in the peripheral blood will be measured at baseline (week 0) and weekly thereafter (till ICU discharge). Total DNA from the blood sample will be extracted from the buffycoat using a DNA Analysis Kit (Machery Nagel Blood XL, Germany) and mtDNA quantity will be analysed using SYBR Green Master Mix Real Time PCR kit (Applied Biosystems, Warrington, United Kingdom). Triplicate analysis of blood samples will be conducted and trends of longitudinal increases in mitochondrial DNA levels will be reported.

#### Illness Severity

Blood lactate levels will be measured before intervention and 30 minutes post intervention with arterial blood using a standard benchtop Arterial Blood Gas Analyser (ABL 700 Series gas Machines, RADIOMETER, Copenhagen, Denmark). Lactate concentration trends will be used to analyse lactate clearance rates [71] as a marker of illness severity.

#### Muscle oxygenation and Microcirculation

Muscle oxygenation and microcirculatory changes will be measured using the Near Infrared Oxygenation (NIR0) Monitor and the Orthogonal Polarization Spectral (OPS) Microscan respectively.

Muscle (tissue) oxygenation will be measured pre and post intervention daily using the NIRO (NIRO-200, Hamamatsu, Japan) with a probe placed on a peripheral muscle to detect levels of muscle oxygenation (StO_2_) following acute exercise [72]. Muscle oxygen consumption will be further analysed through induced ischemia and active hyperaemia [73]. A pneumatic cuff will be placed above the elbow and inflated to 50 mmHg above the patient's systolic blood pressure and the occlusion will be retained for 3 minutes inducing local ischemia. StO_2 _will be recorded continuously for 3 minutes before, during and after arterial occlusion [44].

Improvements in microcirculation following acute exercise will be measured pre and post intervention session once weekly using the OPS MicroScan (Microvision Medical Inc, Wallingford, PA, USA) with a non-invasive probe placed in the sub-lingual space to detect functional capillary density and blood flow velocity as indicators of microcirculatory function [74] using video images. Five video sequences per patient will be graded by 3 independent observers and assigned a flow velocity score to each of the 4 quadrants of each image [75]. Capillary density will be calculated using superimposed calibrated grid of vertical and horizontal lines on the images of number of small (< 20 μm) vessels crossing the lines of the grid divided by the total length of the lines, yielding the number of small vessels per millimetre [76]. An average of these 5 readings will be calculated for discrete capillary density value for each time point at which imaging was performed.

### Confounding Variables

Critical illness neuropathy and myopathy is a possible feature of critically ill patients. Formal tests to establish critical illness neuropathy or myopathy can be painful, invasive, time consuming and expensive [6]. Such formal diagnosis may not implicate any pertinent findings in this trial. Basic physiotherapy assessment muscle testing done as part of regular intervention assessment using the Medical Research Council or Manual Muscle Test will detect any possible development of weakness without requirements for formal diagnosis and is accepted as configuration of critical illness polyneuromyopathy [77].

### Sample size

Clinically important difference and the standard deviation estimates used in our sample size calculations were based on a previous clinical trial [64]. Sample size was calculated using the ACIF [62] for hypothesis testing with a type I error rate of 0.05 and 0.025 with Bonferroni adjustment and type II error rate of 0.20 (80% power). A minimum of 35 per group (70 total) is required to detect a minimum clinical difference (effect size) of 0.7 for physical functional outcomes which will yield clinically significant results for the main hypothesis achievable by intervention. Projection of the sample size after adjusting for attrition or withdrawal rates, participation refusals and possible death due to sepsis; based on admission and mortality rates in the RBWH ICU, 126 patients per group (252 in total) will be required.

A smaller number of patients will undergo mtDNA, tissue oxygenation and microcirculatory measurements as these will be tested as pilot investigations. To test these secondary hypotheses, a minimum of 58 per group is required to detect a minimum clinical difference of 0.8 for clinically significant changes in mitochondrial DNA [78], a minimum 91 per group is required to detect a clinical difference of 0.4 and 59 per group is required to detect a minimum clinical difference of 0.7 for clinically significant changes in tissue oxygenation [45] and microcirculation [46] respectively, all corrected for with Bonferroni adjustment of 0.025.

### Data Management and Statistical Analysis

A baseline comparison using both student t-tests and chi-square for equal proportions of demographic data will be done between groups at enrolment. Data will be analyzed using SPSS Version 17.0. The distribution, range of scores and heterogeneity will be examined for participants in both groups. Mean changes scores, standard deviations and 95% confidence intervals will be calculated and specific analysis detailed under each outcome measure will be performed. Repeated measures, between/within analysis of variance (ANOVA) will be performed to investigate for the main effects of time and group. Post hoc analyses will then be performed to ascertain where the differences occur. A Bonferroni correction will be used as the post hoc measure as there are multiple outcome measures. Analysis will be by both intention-to-treat and per-protocol method. Any violations of the protocol will be noted. Statistical significance will be set at p < 0.05 and p < 0.025(two-tailed). All efforts will be taken to avoid missing data but if it occurs a carry forward imputation will be done and linear mixed modelling will be used for overall analysis. Reasons for loss of follow up will be recorded.

## Discussion

The i-PERFORM Trial is an original study in investigating early rehabilitation in patients presenting with sepsis syndromes. Fully powered to be a larger trial from the pilot study, this RCT has been modified to include detailed and intensive interventional measures and further outcome measures; SF-36, PFIT, blood lactate, mitochondrial DNA, muscle oxygenation and microcirculation dynamics.

Controversies still exist with regards to the implementation of early exercise in critically ill patients despite growing evidence in the literature [7,79]. Introducing early rehabilitation in patients with sepsis syndromes is challenging and the direct physiological implications are unknown. Novel findings from the i-PERFORM Trial involving physiological markers of oxidative stress and microcirculation will translate better understanding of the short-term systemic effects of early exercise in critical illness. The mechanisms and associations between early exercise and the inflammatory effects of sepsis explored in this trial will implicate on future rehabilitation management of patients with sepsis in intensive care units.

## Key Messages

• The i-PERFORM RCT will determine whether early targeted rehabilitation will achieve a higher functional level and an improved quality of life in patients with sepsis.

• Participants in the intervention arm will receive early, targeted, individualised rehabilitation program comprising of passive, active and resisted exercises, electrical stimulation to major lower limb muscles, sitting out of bed with exercises and tilt-tabling to improve orthostatic reflexes, ergometry exercises for upper and lower limbs and ambulation.

• Physical functional capacity, quality of life, psychological wellbeing, inflammatory biomarkers, oxidative stress markers, illness severity, muscle oxygenation and microcirculation will be assessed using standard instruments at specific times before, during and after exercise sessions.

• Short-term systemic findings from early rehabilitation in sepsis will be novel and will translate better understanding of the acute effects of early exercise in critical illness.

## Abbreviations

**ACIF**: Acute Care Index of Function; **ANOVA**: Analysis of Variance; **ANZCTR**: Australian New Zealand Clinical Trials Registry; **APACHE II**: Acute Physiological and Chronic Health Evaluation II; **ATP**: Adenosine TriPhospate; **BIS**: Bioelectrical Impedance Spectroscopy; **CONSORT**: Consolidated Standards of Reporting Trials; **CV**: Coefficient of Variation; **HADS**: Hospital Anxiety and Depression Scale; **ICU**: Intensive Care Unit; **IL-6**: Interleukin-6; **IL-10**: Interleukin-10; **mtDNA**: Mitochondrial DNA; **NIRO**: Near Infrared Oxygenation; **OPS**: Orthogonal Polarisation Spectral Microscan; **PCR**: Polymerase Chain Reaction; **PFIT**: Physical Function ICU Test; **PBMC**: Peripheral Blood Mononuclear; **RBWH**: Royal Brisbane and Women's Hospital; **RCT**: Randomised Controlled Trial; **ROS**: Reactive Oxygen Species; **SF-36**: Short Form-36 Health Questionnaire; **SOFA**: Sequential Organ Failure Assessment; **StO_2_**: Tissue/Muscle Oxygenation; **TNF-α**: Tumour Necrosis Factor alpha.

## Competing interests

The authors declare that they have no competing interests.

## Authors' contributions

All authors contributed to the study design and methods, and the development of the grant application. JDP specifically contributed to initial conception of the project, pilot data and exercise strategies. GK and RJB contributed to the extended concept of the project for secondary measures. JDP and GK contributed to the statistical methods and power calculations. GK drafted the manuscript and all other authors critically revised it for important intellectual content. All authors approved the final version of the manuscript for publication.

## Authors' information

**Ms Geetha Kayambu**, BSc Phyt (Hons), Physiotherapist, PhD Candidate

The University of Queensland, School of Medicine, Burns Trauma & Critical Care Research Centre, Brisbane QLD 4029, Australia

Contact Address:

The Royal Brisbane and Women's Hospital, Level 7, Block 6, Herston, Brisbane QLD 4029, Australia

**Associate Professor Robert J Boots**, MBBS (Hons), PhD, MMedSci_Clin Epi_, MHAIS, FRACP, FJFICM

Deputy Director

The Royal Brisbane and Women's Hospital, Department of Intensive Care Medicine, Burns Trauma & Critical Care Research Centre, Brisbane QLD 4029, Australia

**Dr. Jennifer D Paratz**, FACP, Mphty, PhD, Grad Cert Ed (Medical and Health Sciences)

Research Fellow and Chair

Burns Trauma & Critical Care Research Centre, The University of Queensland, School of Medicine, Brisbane QLD 4029, Australia

## Pre-publication history

The pre-publication history for this paper can be accessed here:

http://www.biomedcentral.com/1471-2253/11/21/prepub
